# Systematic Review of Brain-Eating Amoeba: A Decade Update

**DOI:** 10.3390/ijerph20043021

**Published:** 2023-02-09

**Authors:** Mohd ‘Ammar Ihsan Ahmad Zamzuri, Farah Nabila Abd Majid, Massitah Mihat, Siti Salwa Ibrahim, Muhammad Ismail, Suriyati Abd Aziz, Zuraida Mohamed, Lokman Rejali, Hazlina Yahaya, Zulhizzam Abdullah, Mohd Rohaizat Hassan, Rahmat Dapari, Abd Majid Mohd Isa

**Affiliations:** 1 Negeri Sembilan State Health Department, Jalan Rasah, Seremban 70300, Malaysia; 2 Department of Psychiatry, Hospital Canselor Tuanku Muhriz, Faculty of Medicine, National University of Malaysia Jalan Yaacob Latif, Bandar Tun Razak, Cheras, Kuala Lumpur 56000, Malaysia; 3 Public Health Division, Ministry of Health Malaysia, Putrajaya 62000, Malaysia; 4 Department of Community Health, Faculty of Medicine, National University of Malaysia Jalan Yaacob Latif, Bandar Tun Razak, Cheras, Kuala Lumpur 56000, Malaysia; 5 Borneo Medical and Health Research Centre, Faculty of Medicine and Health Sciences, Universiti Malaysia Sabah, Kota Kinabalu 88400, Malaysia; 6 Department of Community Health, Universiti Putra Malaysia, Serdang 43400, Malaysia; 7 Faculty of Education and Liberal Arts, INTI International University, Persiaran Perdana BBN Putra Nilai, Nilai 71800, Malaysia

**Keywords:** brain-eating amoeba, *Naegleria fowleri*, primary meningoencephalitis, encephalitis, amoebic encephalitis, free-living amoeba

## Abstract

Introduction: Primary amoebic meningoencephalitis (PAM) is a rare but lethal infection of the brain caused by a eukaryote called *Naegleria fowleri* (*N. fowleri*). The aim of this review is to consolidate the recently published case reports of *N. fowleri* infection by describing its epidemiology and clinical features with the goal of ultimately disseminating this information to healthcare personnel. Methods: A comprehensive literature search was carried out using PubMed, Web of Science, Scopus, and OVID databases until 31 December 2022 by two independent reviewers. All studies from the year 2013 were extracted, and quality assessments were carried out meticulously prior to their inclusion in the final analysis. Results: A total of 21 studies were selected for qualitative analyses out of the 461 studies extracted. The cases were distributed globally, and 72.7% of the cases succumbed to mortality. The youngest case was an 11-day-old boy, while the eldest was a 75-year-old. Significant exposure to freshwater either from recreational activities or from a habit of irrigating the nostrils preceded onset. The symptoms at early presentation included fever, headache, and vomiting, while late sequalae showed neurological manifestation. An accurate diagnosis remains a challenge, as the symptoms mimic bacterial meningitis. Confirmatory tests include the direct visualisation of the amoeba or the use of the polymerase chain reaction method. Conclusions: *N. fowleri* infection is rare but leads to PAM. Its occurrence is worldwide with a significant risk of fatality. The suggested probable case definition based on the findings is the acute onset of fever, headache, and vomiting with meningeal symptoms following exposure to freshwater within the previous 14 days. Continuous health promotion and health education activities for the public can help to improve knowledge and awareness prior to engagement in freshwater activities.

## 1. Introduction

Amoebic encephalitis, an infection of the central nervous system, is extremely uncommon but often lethal. Both primary amoebic meningoencephalitis (PAM) and granulomatous amoebic encephalitis are types of amoebic encephalitis that can afflict people [1]. Amoebic encephalitis is caused by free-living amoebae (FLA), which are microorganisms found naturally in freshwater environments, such as lakes and rivers. However, FLA do not need a specific host to maintain their viability [2]. Four primary genera of amoebae are responsible for disease transmission in humans: *Naegleria*, *Acanthamoeba*, *Sappinia*, and *Balamuthia* [3]. The former is known to cause PAM.

PAM is characterised as an infection that rapidly spreads across the central nervous system, resulting in the loss of brain tissue and significant brain oedema. Although 47 different species of *Naegleria* are known to date, only *N. fowleri* is responsible for PAM [4]. *N. fowleri*, also known as a brain-eating amoeba, was named after Malcolm Fowler, who described the first PAM infection in Australia [5]. The organism is thermophilic, thriving in freshwater at high temperatures, especially that contaminated with soil [6]. Its life cycle has three stages—cyst, trophozoite, and flagellate—and it can alternate these given specific conditions. It enters humans in the trophozoite form [7].

Once *N. fowleri* enters the body, the onset of disease can be rapid, ranging from 2 to 8 days but sometimes quicker [8]. Early symptoms on presentation are usually vague, with common complaints of fever and headache. As the disease progresses and more brain tissue is affected, neurological signs develop [9]. At this time, diagnosis remains a challenge because it mimics bacterial meningitis [1,10]. A thorough history taking, including a significant exposure to freshwater before symptom onset, can increase the likelihood of achieving the probable diagnosis. An analysis of cerebrospinal fluid (CSF) following neurological manifestations increases the chance of an accurate diagnosis. The application of neuroimaging techniques, such as magnetic resonance imaging (MRI) and computer tomography (CT), can improve the visualisation of various affected regions of the brain, especially in the later stages of the disease [11,12].

The possible reservoirs of *N. fowleri* are extensive [13]. A recent meta-analysis estimated that the pooled prevalence of *Naegleria* spp. in water sources across 35 countries was 26.42% (95% CI = 21.52–31.63; [14]). Although Malaysia, a warm and temperate country located on the equator, has not recorded a case of *N. fowleri*, a few local studies have shown a significant abundance of *Naegleria* spp. [15]. This may become a public health threat because part of the country is still battling with other waterborne parasitic infections [16]. More than 70% of water samples from 11 states harboured *Naegleria* spp. [17].

As the movement restrictions imposed during the COVID-19 pandemic have slowly eased, travelling and water activities have begun to resume. Unexpectedly, the recent announcement of a brain-eating amoeba case reported in South Korea following a history of travelling to a Southeast Asian country has sparked the need to re-highlight this illness. A recent systematic review [18] on *N. fowleri* described cases before the COVID-19 pandemic until 2018. Therefore, this systematic review aimed to consolidate the epidemiological and clinical characteristic knowledge of *N. fowleri* infection gathered in the last decade. The findings obtained can shed new light on the latest infection updates and allow clinicians and public health medicine specialists to be more informed and plan appropriate interventions.

## 2. Methods

The systematic review was conducted using four databases: PubMed, Web of Science, Scopus, and OVID. The selected databases were chosen given the authors’ familiarity and subscription coverage by the institution, and they are renowned for their large collections. The search was performed in accordance with the Preferred Reporting Items for a Systematic Review and Meta-analysis (PRISMA) checklist [19]. The following keywords were applied:

“amebiasis” OR “amoeba” OR “protozoa” OR “brain-eating amoeba”

AND

“*Naegleria fowleri*” OR “*Naegleria*”

AND

“nerve system” OR “brain” OR “spinal cord” OR “central nervous system”

AND

“meningoencephalitis” OR “brain infection” OR “primary amoebic meningoencephalitis”

### 2.1. Selection Criteria

**Figure 1:**
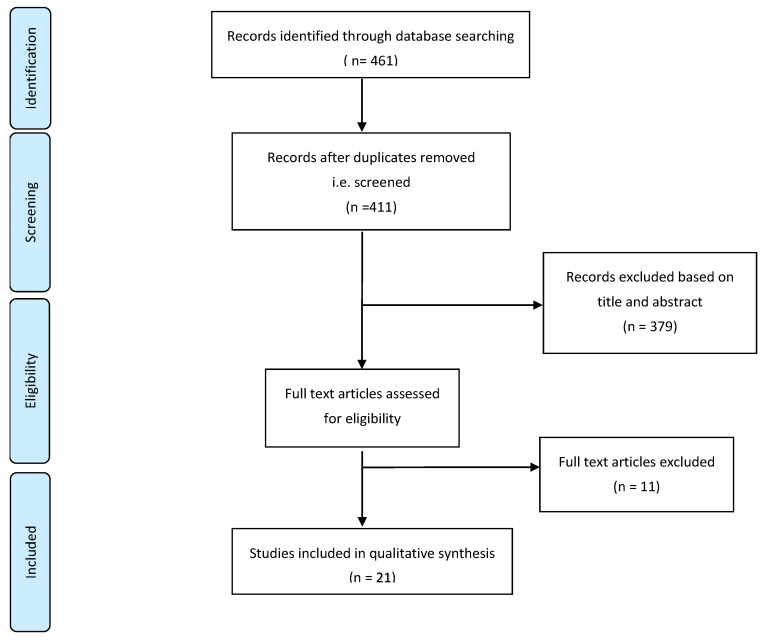
The flow diagram of the process of study selection.

The target population of this search was all patients with a diagnosis of *N. fowleri*, regardless of whether the diagnosis was at the initial presentation or confirmed at a later stage of disease progression, including through autopsy. The exclusion criteria of this search were as follows: (a) letters to the editor; (b) systematic and narrative review paper articles; (c) physiology papers; (d) animal studies; (e) non-English articles; and (f) papers on other types of amoebic infection, including *Acanthamoeba* spp. and *Balamuthia* spp. To minimise the risk of not including the targeted articles, a meticulous search of papers from 1965 to 2022 was conducted automatically. Subsequently, the main author manually filtered out all the articles published before 2013, counter-checked by two more independent authors. The year 1965 was selected because it saw the first published case of confirmed *N. fowleri* [5]. These articles were identified through the title screening process. The articles’ abstracts were then screened using the eligibility criteria. Full-text articles were subsequently included in the qualitative synthesis. A flow diagram of the article search is described in Figure 1.

### 2.2. Operational Definition

*Naegleria fowleri* infection is also known as brain-eating amoeba. The criteria used in this paper limited articles to confirmed cases of *N. fowleri* infection, as well as concurrent infection with other diseases. The latter was included to account for the limitations faced by facilities in diagnosing *N. fowleri* infection. The method for diagnosing *Naegleria* infection included but was not limited to using a CSF sample, also extending to blood and biopsy samples.

### 2.3. Data Extraction Tool

All researchers independently extracted the information from each article into an Excel sheet. The data extracted were (a) the year of publication, (b) country, (c) the number of cases, (d) gender, (e) the age of the patient, (f) ethnicity, (g) comorbidities, (h) risk factors, (i) exposure, (j) symptoms, (k) time to onset, (l) signs elicited, (m) full blood count, (n) other blood results, (o) CSF result, (p) PCR result, (q) culture result, (r) imaging result, (s) initial diagnosis, (t) treatment instituted, and (u) survival outcome. A second reviewer was employed to cross-check the articles assigned and provide comments in the table.

### 2.4. Quality Assessment Tool

By using the Joanna Briggs Institute (JBI) Critical Appraisal Checklist for Case Reports, all the articles were critically appraised for their quality by at least two independent reviewers. Unlike other study designs, this is probably sufficient to optimally assess articles of this design [20]. Additionally, to ensure rigour, Cohen’s kappa analysis was also conducted between the result of the two reviewers. The obtained result was 0.90, thus showing good agreement [21]. Nevertheless, any discrepancy about the quality was resolved by employing a third reviewer.

## 3. Results

### Study Selection and Characteristics

A total of 461 articles were found during the initial search of the four large databases; 50 duplicated articles were removed, and 379 articles were excluded based on the title and abstract screening. From this initial screening, articles with a title or abstract focusing solely on the physiology context and narrative reviews were removed from the list; 32 articles underwent full manuscript review. Ultimately, 21 articles were accepted for qualitative synthesis in this systematic review. The most common reasons for article rejection were being a physiology paper, narrative review, animal study, or in vitro study and covering different FLA.

Table 1 shows the studies included. Of the 21 articles included, 1 reported a case series (two cases; [22]. Therefore, this study presented a total of 22 cases of *N. fowleri* infection for discussion. Of all the studies included, seven were from the USA: [23,24,25,26,27,28]. Four studies were from India: [29,30,31,32]. Two studies were from China: [33,34]. The remaining studies were from Australia [35], Bangladesh [36], Nepal [37], Norway [38], Pakistan [39], Taiwan [40], Turkey [41], and Zambia [42]. A total of 16 (72.7%) patients included in this study died.

Patients’ ages were heterogeneous, ranging from 11-day-old neonates [41] to a 75-year-old [40]. The majority of the cases were male (n = 15, 68.2%) and of United States nationality (n = 5, 22.7%). Of the studies, 18 reported a previous history of water contact activities, such as swimming at a lake, river, or waterpark. Only two studies reported no previous untreated water contact [32,37], and one study did not address this risk factor [39]. Another notable factor associated with an increased risk of disease transmission was irrigating the nostrils, highlighted by McLaughlin and O’Gorman (2019) [35], Sazzad et al. (2020) [36], and Stubhaug et al. (2016) [38].

Overall, the majority of the symptom onset occurred in less than 14 days, with some studies reporting it to be as early as 2 days [42] or 3 days [28,34] following exposure. The most common symptom reported was fever (n = 19, 86.4%), followed by headache (n = 16, 72.7%) and vomiting (n = 14, 63.6%). The most common neurological symptom was convulsion, occurring in nine cases (39.1%). There were varying presentations of convulsion: some presented it early in the course of the disease, whereas others presented it as late sequelae. Three cases (13.0%) reported limb weakness [31,37,40]. For paediatric cases aged less than 1 year, all patients had a fever and reduced oral intake.

In terms of blood work-up, the commonest finding was leucocytosis and raised blood C-reactive protein (CRP). However, one study observed a case to have concurrent microcytic hypochromic anaemia [32], and another from India reported a raised erythrocyte sedimentation rate (ESR) [30]. All but one study reported CSF result findings [24]. The most common findings were low glucose levels with concomitant high protein levels. The opening pressure was high, and the CSF was turbid. The CSF finding showed a high level of white cells.

PCR tests were carried out in all but four studies [26,29,30,37]. The sample sent for the PCR analysis was commonly from a CSF sample. Some of the studies employed culture and sensitivity tests to confirm the diagnosis, mostly unsuccessfully. Imaging techniques, such as non-contrast CT, contrast-enhanced CT, and MRI, were carried out in most cases. Finally, cases that achieved a confirmatory or probable result for *N. fowleri* infection before death were treated with antifungal medications appropriately. One case was diagnosed as PAM at autopsy using immunohistochemistry [23].

## 4. Discussion

This study consolidated and updated the current knowledge on brain-eating amoebae. The rare occurrence of *N. fowleri* infection has resulted in a few extra case reports over a decade. This is in line with previous reports from the USA using data from 1978 until 2018, in which the incidence of PAM associated with water activities ranged between 0 and 6 cases per year (n = 85 cases in total; [43]). However, certain geographical areas may possess a slightly higher risk for transmission to occur, as evidenced by four more cases in 2022 reported in Pakistan alone [44]. Regardless of transmissibility, measuring the true burden of the infection remains difficult, as highlighted in previous papers that concluded that officially reported cases are typically an underestimation [45,46].

The challenges in diagnosing PAM in the clinical setting might have contributed to the underestimation of the infection. The initial vague presentation and subsequent presentation that mimics bacterial meningitis may prevent clinicians from making the correct diagnosis [47]. The rarity of its occurrence may cause further delays. Likewise, the limitations in diagnostic tools for detecting the culprit organism could make the matter worse [48]. For instance, directly visualising the presence of *N. fowleri* in a sample is highly operator-dependent and can be less sensitive with untrained personnel. Analyses using PCR techniques might not be readily available in some centres, especially in rural and district hospitals [49]. Although FLA cultures require specific media and take a longer time to complete, a negative test result may still requires additional analysis as evidence to support correlations.

Based on the consolidated findings of the included cases, we propose several key features to aid clinicians who may encounter patients with similar presentations. The illness typically presents acutely with fever associated with headache and vomiting. The presence of meningeal symptoms following contact exposure to freshwater warrants immediate testing to confirm the diagnosis. Baseline blood parameters, such as full blood counts and renal and liver function tests, may not show large deviations. However, general infective markers, such as ESR and CRP, will always be elevated. Hence, interpreting the initial results promptly and meticulously is important to come closer to an accurate diagnosis.

A high clinical index of suspicion remains fundamental to achieving the earliest diagnosis of PAM considering the diagnostic tools available. Hence, healthcare personnel must be able to elicit the travel history to where the amoeba is found and, more crucially, any significant exposure to warm freshwater [50]. *N. fowleri* has been identified in almost every continent, except for Antarctica [51]. The amoeba’s thermophilicity and ubiquity mean that it can be discovered in a wide variety of environments, including natural hot springs, ponds, rivers, freshwater lakes, drinking water distribution systems, untreated swimming pools, fountains, hospitals, thermal waters, untreated drinking water, and waterparks [52,53]. The success of determining such vital contact exposure should warrant immediate work to identify the presence of *N. fowleri* because PAM can progress rapidly, leading to death within just a few days.

The onset is rapid because its mechanism of entry is direct to the brain. Following water contact activities, *N. fowleri* enters the nasal cavity, attaches to the nasal mucosa, burrows into it, and then travels through the cribriform plate and onwards along the olfactory nerves to the olfactory bulb. The aftermath of brain penetration leads to brain swelling, the herniation of the cerebral artery, and, ultimately, death [7,54]. The pathogenicity of *N. fowleri* and the intensity of the host immune response correlate with the severity of central nervous system symptoms [50]. Two proposed models have been developed to elucidate the pathogenesis of PAM: contact-dependent mechanisms and contact-dependent mechanisms. The former is attributed to adhesion and phagocytic food cups, and the latter is related to the cytolytic molecules secreted by *N. fowleri* [55].

The symptoms exhibited by the infection correspond to both the immune response and areas affected in the brain. Constitutional symptoms, such as fever, chills, and lethargy, occurred almost in all cases reported in the literature as a result of the production of reactive oxygen species that subsequently activate the epidermal growth factor receptor pathway and induce the expression of MUC5AC (mucin), as well as pro-inflammatory cytokines, such as interleukin 8 (IL-8) and cytokine IL-1β [56,57]. Different parts of the brain that are affected yield more specific signs and symptoms. Hence, neuroimaging techniques may help to determine the focal area involved.

CSF sample results may help to rule out the viral aetiology of central nervous system involvement. Typically, a lumbar puncture can be performed when neurological symptoms exist and when the opening pressure is low [58]. Low CSF glucose levels with concomitant high levels of CSF proteins exclude viral causes. Subsequently, a direct visualisation of the CSF sample can be carried out if the laboratory has the expertise to detect motile amoebae under light microscopic observation [59]. We recommend that the sample be analysed in an accredited laboratory to prevent the risk of a false positive result on microscopy. This is because host cells, such as leukocytes and macrophages, can mimic amoeba trophozoites and, thus, contribute to a false positive result [60]. As such, diagnosing PAM is better achieved using molecular techniques, as recommended by the US CDC, on a CSF sample or brain tissue yielding *N. fowleri* [61]. An accurate diagnosis is critical because aggressive treatment can be commenced rapidly to ensure survival.

Given the high fatality associated with *N. fowleri* infection, extensive public health measures must be taken. First, the public needs to be educated about this illness through a continuous health awareness program. Health promotional activities should include consistent reminders about protective habits for those going to freshwater contact events. The information should be disseminated at regular intervals and more focused on high-risk areas. Among the messages to be included are the following:

- Do not engage in water-based activities near warm, stagnant water, especially if the water is shallow, low, and has a weak flow rate;
- Pinch the nose or use a nose clip when taking part in water-related activities in potentially contaminated water;
- Keep the head above the water level when swimming in freshwater, hot springs, and other untreated thermal bodies of water;
- Avoid diving and jumping into stagnant freshwater;
- Do not to dig or stir up sediment from the bottom of bodies of water;
- Try to avoid water-related activities if there are any open cuts or wounds on the body.

Health education messages must also be widely circulated to encourage the public to seek immediate treatment when starting to develop symptoms after freshwater-based activities. Those who own swimming pools, spas, and hot tubs are required to disinfect and properly maintain them. Finally, the public should be encouraged to use boiled, filtered, or sterile water for nasal or sinus irrigation instead of raw tap water.

This study has several limitations. The first is the limitation of diagnosing PAM itself, which likely resulted in unrecognised cases and underreporting to health officials. As such, this study likely accounts for a small fraction of the real numbers of *N. fowleri*. Secondly, only English-language texts were included, although the most recent case occurred in a non-primarily English-speaking country. Finally, not all full texts could be acquired because some journals are not subscribed to by the authors’ institution.

## 5. Conclusions

*N. fowleri* infection is rare but leads to PAM. Its occurrence is worldwide, with a significant risk of fatality. This study consolidated the most recent publications from the last decade featuring both epidemiology and clinical presentation. The suggested probable case definition in accordance with the findings includes the acute onset of fever, headache, and vomiting with meningeal symptoms following exposure to freshwater within the previous 14 days. The use of a broad probable clinical definition will ensure an early index of suspicion during medical consultation, allowing for the prompt commencement of diagnostic investigations. A direct visualisation of the amoeba from CSF samples under microscopy is possible but can lead to false positive results. The use of molecular techniques is recommended for confirmatory tests. Ultimately, achieving an early accurate diagnosis is vital to instituting aggressive treatment.

## Figures and Tables

**Table 1:** Epidemiological, clinical, and laboratory data of the PAM cases reviewed.

No	Citation	Country	Age (Year)	Sex	Exposure	Symptoms	Time to Onset	Full Blood Count	CSF Result	PCR Result	Survival
1	Chen et al., 2019 [33]	China	43	Male	Waterpark activities; traveller	Fever; myalgia; fatigue; persistent occipital headache (for 2 days)	5 days	Leucocytosis; CRP	Turbid; high pressure; glucose low; leucocyte high; protein high; Pandy’s test positive; trophozoites of *N. fowleri* with Wright–Giemsa stain	*N. fowleri* positive	Mortality
2	Celik and Arslankoylu 2021 [41]	Turkey	11-day-old	Male	History of bathing with unchlorinated well water	Fever; inability to suck; irritability; convulsion (for 2 days)	4 days	Leucocytosis; CRP	Leucocytosis; protein high; glucose low; negative for microbial growth, TORCH, acid-resistant bacilli, Ziehl–Neelsen staining	*N. fowleri* positive	Mortality
3	Ravinder et al., 2016 [31]	India	15	Male	Bathing in unclean village pond	Fever; vomiting; left-sided body weakness; convulsion; sudden loss of consciousness; jerky bilateral hand movement	No data	Leucocytosis	Direct microscopy—flagellated parasite and spherical budding yeast cells; latex agglutination test was positive for Cryptococcal antigen	Not performed	Survive at point of time
4	Chomba et al., 2017 [42]	Zambia	24	Male	Swimming in river; police recruit at training camp	Fever; convulsion	2 days	No data	No bacterial or fungal pathogens were detected in CSF day 1 and day 3; numerous highly motile amoebic trophozoites and cysts day 8	*N. fowleri* positive	Mortality
5	Stowe et al., 2017 * [22]	USA	4 and 14	Male	(1) Camping and swimming at lake	(1) Fever; convulsion; headache, vomiting; difficulty ambulating; altered mental status; (2) generalised muscle weakness; tactile fever; vomiting; confusion; convulsion	Both 8 days	No data	Grossly abnormal; (1) free-swimming amoebae	*N. fowleri* positive	Mortality
6	McLaughlin and O’Gorman 2019 [35]	Australia	56	Male	Swimming in stagnant water that was also accessed by cattle; irrigating nostrils	Headache; photophobia; nausea; vomiting; neck stiffness	No data	No data	Turbid; glucose low; protein high; leucocytosis; free-living amoebae	*N. fowleri* positive	Mortality
7	Cope et al., 2018 [27]	USA	18	Female	Freshwater swimming; water rafting activities	Fever; headache; lethargy	14 days	No data	High pressure; leucocytosis; glucose low; protein high; wet mount of the CSF revealed possible motile trophozoites	*N. fowleri* positive, with concomitant detection of *Balamuthia mandrillaris* and *Acanthamoeba* spp.	Mortality
8	Sazzad et al., 2020 [36]	Bangladesh	15	Male	Bathing and contact with untreated ground water and river; irrigating nostrils	Fever; generalised headache; vomiting; weakness; neck stiffness; unconscious	No data	No data	Protein high; glucose low; leucocytosis; negative Ziehl–Neelsen stain and acid-fast bacilli	*N. fowleri* positive	Mortality
9	Stubhaug et al., 2016 [38]	Norway	71	Female	Travel to Thailand where hotel was supplied with untreated groundwater well; irrigating nostrils	Nausea; vomiting; fever; fatigue;	No data	Leucocytosis; CRP	Turbid; pressure high; glucose low; protein high; leucocytosis; negative nigrosin, acridine orange stain; equivocal cryptococcal latex antibody	*N. fowleri* positive	Mortality
10	Baral and Vaidya 2018 [37]	Nepal	74	Male	Nil	Fever; global headache; feature of anomic aphasia but no vomiting, seizure, or neurological deficit at presentation; altered sensorium and agitation; gradual weakness of bilateral lower limb and trunks	No data	No data	Leucocytosis; protein high; glucose low; negative for microbial growth, TB, AFB, HSV	Not performed	Mortality
11	Johnson et al., 2016 [24]	USA	21	Female	Swimming in private pool in a desert environment—not being chemically treated. Water supplied to the pool was from a mountain spring	Headache; nausea; vomiting;	14 days	No data	Not mentioned	*N. fowleri* positive	Mortality
12	Huang et al., 2021 [34]	China	8	Male	Swimming in lake	Fever; headache; vomiting; altered consciousness; convulsion	3 days	Leucocytosis; CRP	Leucocytosis; glucose low; protein high; pale, pink, thick necrotic fluid drawn out from the syringe	*N. fowleri* positive	Mortality
13	Mushtaq et al., 2020 [39]	Pakistan	44	Male	Not mentioned	Fever; worsening headache; generalised weakness	No data	Leucocytosis;	Leucocytosis; negative Gram stain, India ink, cryptococcal antigen; positive wet prep for Naegleria	*N. fowleri* positive	Mortality
14	Mittal et al., 2019 [32]	India	8 months	Female	Nil	Fever; chills; rigors; abnormal body movement; vomiting; generalised tonic–clonic seizures; decreased oral intake; decrease urine output	No data	Microcytic hypochromic anaemia; raised CRP	High pressure; protein high; glucose low; leucocytosis; wet mount positive for moving trophozoites of amoeba; negative for India ink, Gram stain	*N. fowleri* positive	Survive at point of time (AOR)
15	Yadav et al., 2013 [29]	India	25-day-old	Male	Untreated water well used for bathing	Fever; reduced feeding; multi-focal seizures (for 10 days)	No data	Leucocytosis; CRP	Leucocytosis; glucose low; protein high; CSF wet mount examination revealed presence of free-living motile amebae	Not performed	Survival
16	Dunn et al., 2016 [25]	USA	12	Female	Swimming at freshwater park	Fever; headache; lethargy; nausea; vomiting	7 days	Leucocytosis	Milky colour; high turbidity; leucocytosis; Giemsa stain positive for *Naegleria* spp.	*N. fowleri* positive	Survival
17	Sood et al., 2014 [30]	India	6	Male	Collateral history—the boy played frequently with water stored in a cement tank used for varied purposes. The water was collected from a nearby diversion channel called “kuhl”	Fever; headache; altered sensorium	No data	Raised ESR	Clear colour; leucocytosis; negative Gram stain, India ink, Ziehl–Neelsen; positive wet mount revealed amoebic and flagellate trophozoites	Not performed	Survival
18	Cope et al., 2015 [23]	USA	4	Male	Water exposure: tap water that was used to supply water to a lawn water slide on which the child had played extensively	Diarrhoea; vomiting; poor oral intake; severe headache; fever; convulsion; lethargy	No data	Leucocytosis	Colourless; pressure high; protein high; leucocytosis; negative Gram stain	*N. fowleri* positive	Mortality
19	Anjum et al., 2021 [28]	USA	13	Male	Swimming at water park—untreated water supply	Headache; fever; intractable emesis; poor oral intake	3 days	Leucocytosis; raised CRP	Pressure high; turbid; glucose low; protein high; leucocytosis; EVD protein high; Wright–Giemsa stain showed amoebic trophozoites	*N. fowleri* positive	Mortality
20	Su et al., 2013 [40]	Taiwan	75	Male	Thermal hot spring	Fever; headache; right arm myoclonic seizure; right-sided limb weakness	No data	Leucocytosis; raised CRP	Turbid; glucose low; protein high; leucocytosis; negative for India ink, Gram stain, AFB, fungi, viral; wet mount smear positive for trophozoites	*N. fowleri* positive	Mortality
21	Heggie and Küpper 2017 [26]	USA	12	Female	Swimming at lake-based water park (shallow depth and sandy bottom)	Fever; vomiting; headache; difficulty waking up from sleep; difficulty holding head up; unable to open eyes; hallucination	Less than 1 week	No data	*N. fowleri*	Not performed	Survival

* The article consists of two cases (a case series). CRP = C-reactive protein.

## Data Availability

Data that are not published require permission from the authors. Please contact the corresponding author for further enquiry.

## References

[1] Pana A., Vini V., Arayamparambil C.A. (2022). Amebic Meningoencephalitis.

[2] Schuster F.L., Visvesvara G.S. (2004). Free-living amoebae as opportunistic and non-opportunistic pathogens of humans and animals. Int. J. Parasitol..

[3] Qvarnstrom Y., da Silva A.J., Schuster F.L., Gelman B.B., Visvesvara G.S. (2009). Molecular confirmation of Sappinia pedata as a causative agent of amoebic encephalitis. J. Infect. Dis..

[4] De Jonckheere J.F. (2014). What do we know by now about the genus *Naegleria*?. Exp. Parasitol..

[5] Fowler M., Carter R.F. (1965). Acute Pyogenic Meningitis Probably Due to *Acanthamoeba* sp.: A Preliminary Report. Br. Med. J..

[6] Maclean R.C., Richardson D.J., LePardo R., Marciano-Cabral F. (2004). The identification of *Naegleria fowleri* from water and soil samples by nested PCR. Parasitol. Res..

[7] Grace E., Asbill S., Virga K. (2015). *Naegleria fowleri*: Pathogenesis, diagnosis, and treatment options. Antimicrob. Agents Chemother..

[8] Visvesvara G.S., Moura H., Schuster F.L. (2007). Pathogenic and opportunistic free-living amoebae: *Acanthamoeba* spp., *Balamuthia mandrillaris*, *Naegleria fowleri*, and *Sappinia diploidea*. FEMS Immunol. Med. Microbiol..

[9] Cooper A.M., Aouthmany S., Shah K., Rega P.P. (2019). Killer amoebas: Primary amoebic meningoencephalitis in a changing climate. JAAPA.

[10] Zahid M.F., Saad Shaukat M.H., Ahmed B., Beg M.A., Kadir M.M., Mahmood S.F. (2016). Comparison of the clinical presentations of *Naegleria fowleri* primary amoebic meningoencephalitis with pneumococcal meningitis: A case-control study. Infection.

[11] Singh P., Kochhar R., Vashishta R.K., Khandelwal N., Prabhakar S., Mohindra S., Singhi P. (2006). Amebic meningoencephalitis: Spectrum of imaging findings. AJNR. Am. J. Neuroradiol..

[12] Ong T.Y.Y., Khan N.A., Siddiqui R. (2017). Brain-Eating Amoebae: Predilection Sites in the Brain and Disease Outcome. J. Clin. Microbiol..

[13] Matanock A., Mehal J.M., Liu L., Blau D.M., Cope J.R. (2018). Estimation of Undiagnosed *Naegleria fowleri* Primary Amebic Meningoencephalitis, United States. Emerg. Infect. Dis..

[14] Saberi R., Seifi Z., Dodangeh S., Najafi A., Abdollah Hosseini S., Anvari D., Taghipour A., Norouzi M., Niyyati M. (2020). A systematic literature review and meta-analysis on the global prevalence of *Naegleria* spp. in water sources. Transbound. Emerg. Dis..

[15] Ithoi I., Ahmad A.F., Nissapatorn V., Lau Y.L., Mahmud R., Mak J.W. (2011). Detection of *Naegleria* species in environmental samples from Peninsular Malaysia. PLoS ONE.

[16] Richard R.L., Ithoi I., Abd Majid M.A., Wan Sulaiman W.Y., Tan T.C., Nissapatorn V., Lim Y.A.L. (2016). Monitoring of Waterborne Parasites in Two Drinking Water Treatment Plants: A Study in Sarawak, Malaysia. Int. J. Environ. Res. Public Health.

[17] Gabriel S., Khan N.A., Siddiqui R. (2018). Occurrence of free-living amoebae (*Acanthamoeba*, *Balamuthia*, *Naegleria*) in water samples in Peninsular Malaysia. J. Water Health.

[18] Gharpure R., Bliton J., Goodman A., Ali I.K.M., Yoder J., Cope J.R. (2021). Epidemiology and Clinical Characteristics of Primary Amebic Meningoencephalitis Caused by *Naegleria fowleri*: A Global Review. Clin. Infect. Dis..

[19] Liberati A., Altman D.G., Tetzlaff J., Mulrow C., Gøtzsche P.C., Ioannidis J.P.A., Clarke M., Devereaux P.J., Kleijnen J., Moher D. (2009). The PRISMA statement for reporting systematic reviews and meta-analyses of studies that evaluate healthcare interventions: Explanation and elaboration. BMJ.

[20] Ma L.-L., Wang Y.-Y., Yang Z.-H., Huang D., Weng H., Zeng X.-T. (2020). Methodological quality (risk of bias) assessment tools for primary and secondary medical studies: What are they and which is better?. Mil. Med. Res..

[21] McHugh M.L. (2012). Interrater reliability: The kappa statistic. Biochem. Med..

[22] Stowe R.C., Pehlivan D., Friederich K.E., Lopez M.A., DiCarlo S.M., Boerwinkle V.L. (2017). Primary Amebic Meningoencephalitis in Children: A Report of Two Fatal Cases and Review of the Literature. Pediatr. Neurol..

[23] Cope J.R., Ratard R.C., Hill V.R., Sokol T., Causey J.J., Yoder J.S., Mirani G., Mull B., Mukerjee K.A., Narayanan J. (2015). The first association of a primary amebic meningoencephalitis death with culturable *Naegleria fowleri* in tap water from a US treated public drinking water system. Clin. Infect. Dis..

[24] Johnson R.O., Cope J.R., Moskowitz M., Kahler A., Hill V., Behrendt K., Molina L., Fullerton K.E., Beach M.J. (2016). Notes from the Field: Primary Amebic Meningoencephalitis Associated with Exposure to Swimming Pool Water Supplied by an Overland Pipe—Inyo County, California, 2015. MMWR. Morb. Mortal. Wkly. Rep..

[25] Dunn A.L., Reed T., Stewart C., Levy R.A. (2016). *Naegleria fowleri* that induces primary amoebic meningoencephalitis: Rapid diagnosis and rare case of survival in a 12-year-old Caucasian girl. Lab. Med..

[26] Heggie T.W., Küpper T. (2017). Surviving *Naegleria fowleri* infections: A successful case report and novel therapeutic approach. Travel Med. Infect. Dis..

[27] Cope J.R., Murphy J., Kahler A., Gorbett D.G., Ali I., Taylor B., Corbitt L., Roy S., Lee N., Roellig D. (2018). Primary Amebic Meningoencephalitis Associated with Rafting on an Artificial Whitewater River: Case Report and Environmental Investigation. Clin. Infect. Dis..

[28] Anjum S.K., Mangrola K., Fitzpatrick G., Stockdale K., Matthias L., Ali I.K.M., Cope J.R., O’Laughlin K., Collins S., Beal S.G. (2021). A case report of primary amebic meningoencephalitis in North Florida. IDCases.

[29] Yadav D., Aneja S., Dutta R., Maheshwari A., Seth A. (2013). Youngest survivor of *Naegleria meningitis*. Indian J. Pediatr..

[30] Sood A., Chauhan S., Chandel L., Jaryal S.C. (2014). Prompt diagnosis and extraordinary survival from *Naegleria fowleri* meningitis: A rare case report. Indian J. Med. Microbiol..

[31] Ravinder K., Uppal B., Aggarwal P., Mehra B., Hasan F., Mridul Daga K. (2016). Co-infection of central nervous system by M. Tuberculosis, Cryptococcus and possibly *Naegleria Fowleri*. Trop. Biomed..

[32] Mittal N., Mahajan L., Hussain Z., Gupta P., Khurana S. (2019). Primary amoebic meningoencephalitis in an infant. Indian J. Med. Microbiol..

[33] Chen M., Ruan W., Zhang L., Hu B., Yang X. (2019). Primary amebic meningoencephalitis: A case report. Korean J. Parasitol..

[34] Huang S., Liang X., Han Y., Zhang Y., Li X., Yang Z. (2021). A pediatric case of primary amoebic meningoencephalitis due to *Naegleria fowleri* diagnosed by next-generation sequencing of cerebrospinal fluid and blood samples. BMC Infect. Dis..

[35] McLaughlin A., O’Gorman T. (2019). A local case of fulminant primary amoebic meningoencephalitis due to *Naegleria fowleri*. Rural Remote Health.

[36] Sazzad H.M.S., Luby S.P., Sejvar J., Rahman M., Gurley E.S., Hill V., Murphy J.L., Roy S., Cope J.R., Ali I.K.M. (2020). A case of primary amebic meningoencephalitis caused by *Naegleria fowleri* in Bangladesh. Parasitol. Res..

[37] Baral R., Vaidya B. (2018). Fatal case of amoebic encephalitis masquerading as herpes. Oxf. Med. Case Rep..

[38] Stubhaug T.T., Reiakvam O.M., Stensvold C.R., Hermansen N.O., Holberg-Petersen M., Antal E.A., Gaustad K., Førde I.S., Heger B. (2016). Fatal primary amoebic meningoencephalitis in a Norwegian tourist returning from Thailand. JMM Case Rep..

[39] Mushtaq M.Z., Mahmood S.B.Z., Aziz A. (2020). A Fatal Case of Primary Amoebic Meningoencephalitis (PAM) Complicated with Diabetes Insipidus (DI): A Case Report and Review of the Literature. Case Rep. Infect. Dis..

[40] Su M.Y., Lee M.S., Shyu L.Y., Lin W.C., Hsiao P.C., Wang C.P., Der Ji D., Chen K.M., Lai S.C. (2013). A fatal case of *Naegleria fowleri* meningoencephalitis in Taiwan. Korean J. Parasitol..

[41] Celik Y., Arslankoylu A.E. (2021). A Newborn with Brain-Eating Ameba Infection. J. Trop. Pediatr..

[42] Chomba M., Mucheleng’anga L.A., Fwoloshi S., Ngulube J., Mutengo M.M. (2017). A case report: Primary amoebic meningoencephalitis in a young Zambian adult. BMC Infect. Dis..

[43] Gharpure R., Gleason M., Salah Z., Blackstock A.J., Hess-Homeier D., Yoder J.S., Ali I.K.M., Collier S.A., Cope J.R. (2021). Geographic Range of Recreational Water-Associated Primary Amebic Meningoencephalitis, United States, 1978–2018. Emerg. Infect. Dis..

[44] Tabassum S., Naeem A., Gill S., Mumtaz N., Khan M.Z., Tabassum S., Naeem R., Mukherjee D. (2022). Increasing cases of *Naegleria fowleri* during the time of COVID 19; an emerging concern of Pakistan. Int. J. Surg..

[45] Panda A., Mirdha B.R., Rastogi N., Kasuhik S. (2020). Understanding the true burden of “*Naegleria fowleri*” (Vahlkampfiidae) in patients from Northern states of India: Source tracking and significance. Eur. J. Protistol..

[46] Yoder J.S., Eddy B.A., Visvesvara G.S., Capewell L., Beach M.J., Panda A., Mirdha B.R., Rastogi N., Kasuhik S. (2020). The epidemiology of primary amoebic meningoencephalitis in the USA, 1962–2008. Epidemiol. Infect..

[47] Heggie T.W. (2010). Swimming with death: *Naegleria fowleri* infections in recreational waters. Travel Med. Infect. Dis..

[48] da Rocha-Azevedo B., Tanowitz H.B., Marciano-Cabral F. (2009). Diagnosis of infections caused by pathogenic free-living amoebae. Interdiscip. Perspect. Infect. Dis..

[49] Madarová L., Trnková K., Feiková S., Klement C., Obernauerová M. (2010). A real-time PCR diagnostic method for detection of *Naegleria fowleri*. Exp. Parasitol..

[50] Siddiqui R., Ali I.K.M., Cope J.R., Khan N.A. (2016). Biology and pathogenesis of *Naegleria fowleri*. Acta Trop..

[51] Maciver S.K., Piñero J.E., Lorenzo-Morales J. (2020). Is *Naegleria fowleri* an Emerging Parasite?. Trends Parasitol..

[52] Güémez A., García E. (2021). Primary Amoebic Meningoencephalitis by *Naegleria fowleri*: Pathogenesis and Treatments. Biomolecules.

[53] Jahangeer M., Mahmood Z., Munir N., Waraich U.-E.-A., Tahir I.M., Akram M., Ali Shah S.M., Zulfqar A., Zainab R. (2020). *Naegleria fowleri*: Sources of infection, pathophysiology, diagnosis, and management; a review. Clin. Exp. Pharmacol. Physiol..

[54] Pugh J.J., Levy R.A. (2016). *Naegleria fowleri*: Diagnosis, Pathophysiology of Brain Inflammation, and Antimicrobial Treatments. ACS Chem. Neurosci..

[55] Sohn H.-J., Song K.-J., Kang H., Ham A.-J., Lee J.-H., Chwae Y.-J., Kim K., Park S., Kim J.-H., Shin H.-J. (2019). Cellular characterization of actin gene concerned with contact-dependent mechanisms in *Naegleria fowleri*. Parasite Immunol..

[56] Cervantes-Sandoval I., de Jesús Serrano-Luna J., Meza-Cervantez P., Arroyo R., Tsutsumi V., Shibayama M. (2009). *Naegleria fowleri* induces MUC5AC and pro-inflammatory cytokines in human epithelial cells via ROS production and EGFR activation. Microbiology.

[57] Kim J.-H., Sohn H.-J., Yoo J.-K., Kang H., Seong G.-S., Chwae Y.-J., Kim K., Park S., Shin H.-J. (2016). NLRP3 Inflammasome Activation in THP-1 Target Cells Triggered by Pathogenic *Naegleria fowleri*. Infect. Immun..

[58] Engelborghs S., Niemantsverdriet E., Struyfs H., Blennow K., Brouns R., Comabella M., Dujmovic I., van der Flier W., Frölich L., Galimberti D. (2017). Consensus guidelines for lumbar puncture in patients with neurological diseases. Alzheimer’s Dement..

[59] Martinez A.J., Visvesvara G.S. (1997). Free-living, amphizoic and opportunistic amebas. Brain Pathol..

[60] Fotedar R., Stark D., Beebe N., Marriott D., Ellis J., Harkness J. (2007). Laboratory diagnostic techniques for Entamoeba species. Clin. Microbiol. Rev..

[61] Centers for Disease Control and Prevention (2022). *Naegleria fowleri*—Primary Amebic Meningoencephalitis (PAM)—Amebic Encephalitis [Internet]. https://www.cdc.gov/parasites/naegleria/diagnosis.html.

